# PROTOCOL: Informal social support interventions for improving outcomes for victim‐survivors of domestic violence and abuse: An evidence and gap map

**DOI:** 10.1002/cl2.1263

**Published:** 2022-06-30

**Authors:** Karen L. Schucan Bird, Nicola Stokes, Carol Rivas, Martha Tomlinson

**Affiliations:** ^1^ Social Research Institute University College London London UK; ^2^ SafeLives Bristol UK

## Abstract

This is the protocol for a Campbell Evidence Gap Map. The main objectives of the EGM are: establish the nature and extent of the primary empirical evidence on informal social support interventions, identify interventions and clusters of evidence suitable for systematic review/evidence synthesis and identify gaps in the evidence on informal social support interventions.

## 1 BACKGROUND

### 1.1 The problem, condition or issue

Data from around the world have highlighted that the Covid‐19 pandemic has intensified domestic violence and abuse (DVA), especially against women and girls (UN Women, 2020). Covid mitigation policies have increased women's vulnerability to DVA through stay‐at‐home mandates alongside wider barriers that impede access to their usual community resources and health care services (Nordhues et al., 2021). During this time, policing, health and frontline services have faced a number of challenges in seeking to identify and support individuals who are/have been experiencing violence and abuse in an intimate relationship. Anecdotal evidence suggests that such victim‐survivors have been less able and/or willing to seek help from traditional first responders (Peterman et al., 2020) whilst more informal sources of support have faced increased demands. Within the UK, Refuge (domestic abuse organisation) saw a 700% increase in the number of visits to their Helpline website during the first ‘lockdown’ in March 2020 (Office for National Statistics, 2020).

DVA can encompass, but is not limited to, various forms of abuse including psychological, physical, sexual, financial and/or emotional abuse from a current or former intimate partner (Home Office, 2013). Victim‐survivors of DVA have identifed the importance of informal social supporters (friends, family, colleagues, or community members) during the pandemic, and beyond. For those ‘living with domestic abuse… the view from outside, from supportive friends, family and neighbours, is so important’ (DVA Survivor, SafeLives unpublished data). Evidence from research suggests that the provision of emotional and tangible support can empower victim‐survivors, and positive responses from family and friends can lead to improvements in their mental and physical health (Coker et al., 2002; L. A. Goodman & Smyth, 2011; Sylaska & Edwards, 2014; Weeks & LeBlanc, 2011). Indeed, informal social support and social networks are widely recognised as playing a key role in enabling disclosure of abuse and facilitating help‐seeking (Morgan et al., 2016; Sylaska & Edwards, 2014). The majority of victim‐survivors eventually disclose to at least one informal supporter (Sylaska & Edwards, 2014), typically following a long period of abuse (SafeLives, 2020). Research suggests that female, young, and marginalised groups are most likely to rely on, and disclose to, informal sources of support. Women are more likely to disclose to informal supporters than men (Sylaska & Edwards, 2014) and younger victim‐survivors express a clear preference for informal rather than formal sources of support (Bundock et al., 2018; Moore et al., 2015; Sylaska & Edwards, 2014). Ethnic and racial minorities are more likely to use informal rather than formal channels of support, pointing to the importance of immediate family and friends for help seeking (Fiolet et al., 2019; Ragavan et al., 2018; Rizo & Macy, 2011). In the UK, Black, Asian and racially minoritised women highlight the importance of informal social networks when mainstream services (e.g., police) are unsupportive (Femi‐Ajao et al., 2020). Socioeconomic status (SES) also influences the use of informal social support with individuals from middle/higher SES more likely to disclose to family or friends than their lower SES counterparts (Sylaska & Edwards, 2014). Informal supporters therefore play a critical function in providing support to victim‐survivors, especially for particular groups.

More widely, informal social support constitutes an important pillar in societal‐wide responses to DVA. Governments in the UK and internationally acknowledge the importance of informal social support for victim‐survivors of DVA (e.g. Home Office, 2019; Australian Institute Of Health And Welfare, 2019; Government of Canada Department of Justice, 2006) although policy initiatives tend to focus primarily on the delivery of support through formal channels (such as criminal justice responses). Within the health and domestic violence sectors, there is growing recognition that practitioners can work to activate and mobilise social support to promote positive and sustained change (Goodman et al., 2016; WHO, 2013). Interventions that promote, enhance, or create informal social support can therefore play a critical role in responding to victim‐survivors of DVA (Sánchez et al., 2020). Yet, very little is known about such interventions.

### 1.2 The intervention

Interventions in DVA are commonly understood to operate on three different levels: primary (preventing the initiation or onset of abuse), secondary (identifying and responding to victim‐survivors) and tertiary (responding to victim‐survivors in the longer term and supporting recovery) (Trabold et al., 2020). This review is principally interested in secondary and tertiary interventions, that is, systematic activities that seek to enhance the informal response to victim‐survivors of DVA (rather than activities that aim to prevent the initiation of abuse). As such, Informal Social Support (ISS) Interventions explicitly target the provision of support to individuals who are/have been experiencing violence and abuse in an intimate relationship. These interventions include ‘systematic activities designed to change the existing quality, level, or function of an individual's personal social network or to create new networks and relationships’ (Budde & Schene, 2004, p. 342). The social networks can be classified into different levels depending on the strength and proximity of the relationship with the victim‐survivor: primary (immediate friends, family, community and work colleagues), intermediate (peers and acquaintances) and tertiary levels (individuals within formal organisations and institutions) (Arón & Lorion, 2003; França et al., 2018). Informal social support interventions are principally focused on the inter‐personal relationships that operate in the primary and intermediate social networks. These interventions explicitly aim to create or enhance informal social support, which can take many forms including emotional, informational and/or practical. Interventions create or enhance informal social support by targeting the providers of the informal social support (such as policies to improve awareness of, and responses to, domestic abuse within the workplace), the victim‐survivor (such as programmes that help victim‐survivors to reconnect with social networks), or the relationship between them (such as guidance for family and friends on how to respond to disclosures of abuse). Interventions can also aim to change the wider community within which informal social support is provided.

Informal social support interventions are understood to work in a number of ways. Interventions can alter the knowledge, attitudes and behaviour of friends, family and wider support networks to improve their awareness and response to domestic abuse. Such ‘informal responses are critical because they wield considerable influence over the trajectory of healing and recovery' (Klein, 2012, p. 115). Interventions can also directly bolster social support for victim‐survivors by leading to changes in the structure, function or dynamics of their social network (França et al., 2018). Strengthened social support is associated with a number of outcomes for victim‐survivors including improved access to resources and help‐seeking behaviours (Kennedy et al., 2012; Liang et al., 2005; Zapor et al., 2018)and mental and physical quality of life‐being (Beeble et al., 2009; Bybee & Sullivan, 2002; Sylaska & Edwards, 2014). Whilst informal social support interventions are, by definition, focused on the provision of support and resources outside of formal services or agencies, there is a growing imperative to formally recognise the potential of such interventions for driving long‐term benefits for victim‐survivors (L. A. Goodman et al., 2016; Sullivan, 2018) and their role within a complex system of responses.

### 1.3 Why it is important to develop the EGM

Informal social support interventions are both numerous and diverse. Around the world, there are multiple examples of informal social support interventions targeting victim‐survivors of DVA. Examples include a global movement #ImamsForShe (that highlights the responsibility of Muslim leaders to address violence against women), Employers Initiative on Domestic Abuse in the UK, and community mobilisation activities in India (Society for Nutrition, Education and Health Action in Mumbai). Yet, there is limited knowledge about the extent of the research on such interventions which is likely scattered across a number of academic sources and ‘grey' literature sources. This EGM serves an important role in consolidating the research evidence in this field, a key purpose of systematic mapping approaches (Gough et al., 2017; Snilstveit et al., 2016). Further, there is limited pooled knowledge about the implementation or effectiveness of these types of interventions, or understanding of the experiences of those who have been involved in the interventions. Therefore, this EGM serves to identify interventions and pockets of research that are fruitful areas for further in‐depth analysis and research synthesis. It serves as a valuable tool in the process of engaging with stakeholders and identifying priorities for further analysis (Gough et al., 2017). Such analysis, together with the EGM, provides a useful resource for practitioners and policy‐makers who may be interested in relevant evidence to inform intervention design and implementation (White et al., 2020).

Concurrently, this EGM serves to highlight the gaps in research knowledge about informal social support interventions. This is important for a number of reasons. First, acknowledging the limitations of the evidence base can serve to pinpoint which interventions are operating in an ‘evidence free’ area (White et al., 2020). Without adequate research knowledge, it may be difficult for practitioners or policy makers to determine how/whether interventions are facilitating appropriate and helpful forms of social support rather that negative responses (which have been associated with adverse outcomes for victim‐survivors, see Femi‐Ajao et al., 2020; Rizo & Macy, 2011; Sylaska & Edwards, 2014). Second, the EGM highlights important research gaps that can be used to inform strategic commissioning of new primary research in the field (White et al., 2020).

## 2 OBJECTIVES

The EGM aims to address the following research question:

What is the nature and extent of empirical research on informal social support interventions for victim‐survivors of DVA?

The main objectives of the EGM are:

- Establish the nature and extent of the primary empirical evidence on informal social support interventions
- Identify interventions and clusters of evidence suitable for systematic review/evidence synthesis
- Identify gaps in the evidence on informal social support interventions.

## 3 METHODS

### 3.1 Evidence and gap map (EGM): Definition and purpose

EGM describe the breadth, purpose and extent of research activity within a given field and/or focus (Gough et al., 2017). Such maps utilise systematic review methods to identify, describe and represent the existing evidence base, serving to highlight the availability and characteristics of such research (Snilstveit et al., 2016). This EGM will undertake the following process, informed by accepted standards of EGM conduct (White et al., 2018): Develop and establish a framework for understanding informal social support interventions (what they are and how they may differ), identify and describe all available empirical research on such interventions, develop a visual representation of the EGM, and analyse the findings to draw inferences for research, policy and practice audiences. Stakeholder engagement will be embedded in this process.

### 3.2 Framework development and scope

Whilst the literature recognises that informal social support interventions vary according to ‘the types and sources of social support they seek to mobilize’ (Budde & Schene, 2004, p. 344), there is no overarching framework for ISS interventions in the field of DVA (Nolet et al., 2020). A framework for the interventions was therefore developed for this EGM. This framework was initially developed by KSB through interaction with two sets of literature: (1) studies of ISS interventions for DVA and (2) research that offered empirical or theoretical contributions to understanding the role played by ISS interventions in leading to change for victim‐survivors. The resulting framework was refined in collaboration with NS, CR and MT The framework was presented to and reviewed by Stakeholders (see ‘Stakeholder engagement’ below).

**Figure 1:**
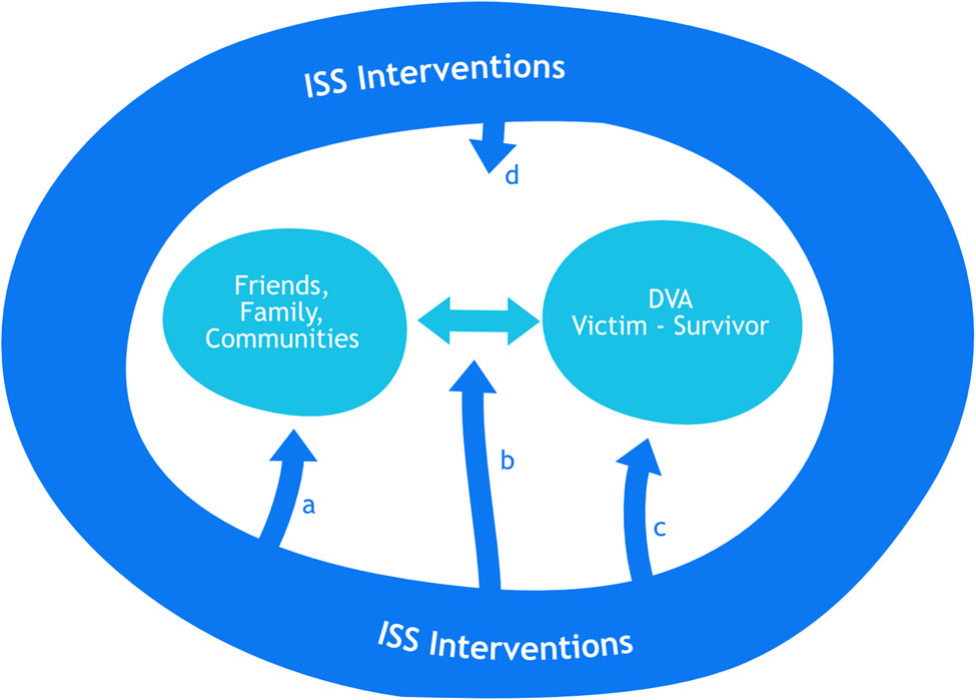
Intervention framework: different types of Informal Social Support Interventions

Figure 1 presents this framework for understanding the nature and range of interventions that aim to create, enhance or facilitate informal social support for DVA victim‐survivors. The relationship between the DVA victim‐survivor and informal supporters (friends, family members and communities) is located at the centre of the Figure. ISS Interventions, represented by the darker arrows, target four different aspects of the relationship and provision of informal social support (as indicated by a, b, c, and d). This includes interventions that focus on *the provider* of informal social support (a), *how* social support is provided and the relationship between the victim‐survivor and informal supporter (b), interventions that focus on *victim‐survivors' ability/resources* to engage with, and utilise, informal social support (c), and interventions that address the *wider community context* within which informal social support is provided (d). The scope of ISS interventions varies and they can include one or more of these (potentially overlapping) foci.

(a) ISS interventions targeting the provider of informal social support. These seek to galvanise existing social support and/or mobilize potential supporters. These types of intervention could include the training of potential informal social supporters (such as clergy in Choi et al., 2019 or peers in Tutty et al., 2017) or new policies aimed at raising awareness about domestic abuse in, for example, the workplace.
(b) ISS interventions that shape the quality of support and/or relationship between informal supporters and DVA victim‐survivors. Such interventions may include the moderation of exchanges between victim‐survivors and supporters in an online forum (e.g., Berg, 2015) or the provision of ‘active strategies of engagement' such as information/brochures aiming to faciliate and influence discussions between victim‐survivors and potential informal supporters (e.g., aimed at hairdressers in Flanigan, 2011).
(c) ISS interventions that focus on victim‐survivors' ability to engage with, and utilise, informal social support. Such interventions include, for example, practitioners assisting victim‐survivors to re‐engage existing networks or develop new forms of social support (Goodman et al., 2016).
(d) ISS interventions that target the community within which the informal social support takes place. Communities are both physical places/spaces and forms of social organisation within which informal support operates (Mancini et al., 2006). These types of intervention include small, specialised community projects to facilitate social support, e.g. local fora for DVA (Wilcox, 2000) or wider initiatives that deliver education and raise awareness about violence and abuse in a community (e.g., community co‐ordinators delivering presentations in key community spaces, such as described in Flanigan, 2011).

### 3.3 Stakeholder engagement

Early and continuing engagement with stakeholders is critical for rapid reviews and EGMs to ensure relevance and actionable outputs (Tricco et al., 2017). An Advisory Group will be created at the outset, composed of stakeholders with lived experience (two people), academic expertise (Alison Gregory, University of Bristol) and policy and/or practice expertise (Andy Myhill, College of Policing; Kate Lawrence Home‐Start East Sussex; Mollin Delve, PHOEBE).

Two meetings will be held to co‐ordinate stakeholder engagement in the EGM. The first meeting will focus on the scope of the review, with online discussions and polling to define the framework (types of informal social support interventions) and scope (type of research, population groups, and outcomes) whilst also feeding into the methods of the EGM (search sources, data extraction). In the second meeting the findings of the EGM will be presented to the Advisory Group to discuss the implications for different audiences.

### 3.4 Conceptual framework

In 2004, Budde and Schene set out an agenda for evaluating ISS interventions, recognising that ‘we are in the early stages of learning how ISS interventions can contribute to preventing…domestic violence' (2004, p. 347). Whilst empirical work on ISS has grown, few studies have explored the role played by ISS interventions, alongside wider DVA service provision, in contributing to social, inter‐ and intra‐personal changes that lead to improvements for DVA victim‐survivors (Goodman et al., 2016; Sullivan, 2018). This section draws on existing research evidence to build an understanding of how informal social support interventions might lead to positive outcomes for victim‐survivors of DVA. This section will consider each type of intervention in turn, as set out in the framework above.

(a) ISS interventions targeting the provider of informal social support.
  Interventions that target current or potential supporters are expected to lead to changes in their knowledge, attitudes and behaviour. These types of interventions may include elements of education, training and/or information provision to generate awareness, dispel myths and build understanding about DVA. Such interventions may target, for example, socio‐cultural norms and gendered assumptions that blame victim‐survivors or minimize their experiences and so hinder informal responses to female victim‐survivors (Mwatsiya and Rasool, 2021). Indeed, it is widely recognised that DVA interventions need to raise awareness and improve understanding of domestic violence (Hester & Westmarland, 2005). Changes in the knowledge and attitudes of informal supporters are expected to improve recognition of abuse and generate helpful responses to victim‐survivors (Edwards & Dardis, 2020; Flecha, 2021). In turn, this improved response is expected to lead to positive outcomes associated with informal social support (see below).
(b) ISS interventions that shape the quality of support and/or relationship between informal support and DVA victim‐survivor.
  The nature of informal supporters' responses to the disclosure of violence and abuse can influence the recovery of the victim‐survivor (Klein, 2012). The literature explains that victim‐survivors identify both negative and positive responses to disclosure. Studies define the provision of emotional support, advice, and/or practical support as positive reactions to disclosures of abuse. Informal supporters who listen and allow the victim‐survivor to talk about the abuse are also helpful (Goodman & Smyth, 2011; Nolet et al., 2021; Sylaska & Edwards, 2014). In contrast, victim‐survivors identify negative responses as refusal of the problem by expressing doubt, blaming the victim, or withdrawing support (Nolet et al., 2020). Research also highlights that the gender and cultural background of the victim‐survivor (and informal supporter) influences the type of response that is considered helpful. Young women, for example, appreciate supporters who listen to them whilst young men welcome physical companionship (McKenzie et al., 2022). There is a body of literature that associates helpful responses with positive outcomes for the victim‐survivor including improvements in quality of life, self‐esteem, autonomy, and mental health (Beeble et al., 2009; Dworkin et al., 2019; Levendosky et al., 2004; Nolet et al., 2021; Sylaska & Edwards, 2014) and promotion of help‐seeking behaviours (Liang et al., 2005). Interventions that target the nature and quality of support provided by informal supporters can therefore seek to maximise helpful responses by promoting an empathetic and accepting reaction (Edwards & Dardis, 2020). Moreover, research also highlights that informal social supporters do not necessarily know how to respond appropriately to disclosure of violence and abuse (Gregory et al., 2017; McKenzie et al., 2022). Interventions can assist informal supporters to provide effective responses and maximise positive outcomes for victim‐survivors (McKenzie et al., 2022).
(c) ISS interventions that focus on victim‐survivors' ability to engage with, and utilise, informal social support.
  Social isolation is key to understanding DVA (Lanier & Maume, 2009) and can constitute a core element of abuse (Flecha, 2021; Vidu et al., 2021). The ongoing process of social isolation (Nolet et al., 2020) means that victim‐survivors are unable to identify anyone, apart from their current or former partner, who they can turn to for help (Lanier & Maume, 2009). This impedes the abilities of victim‐survivors to seek and recieve support from both informal and formal sources (Vidu et al., 2021). Social isolation interacts with wider sociocultural factors such as gendered norms (e.g., expectations about women's role in a family) that may render women particularly vulnerable to the isolating impacts of abuse (Lanier & Maume, 2009). ISS interventions that target victim‐survivor's ability to engage with social networks are therefore critical for enabling individuals to access and recieve helpful forms of support. Evidence suggests that abusive relationships impact the size, quality and strength of a victim‐survivor's social network (Katerndahl et al., 2013; Zaphor et al., 2018). The size of a social network may initially expand after leaving an abusive partner but contract in the longer term (Nolet et al., 2020), and the victim‐survivor may redefine their social relationships in the process (Hydén, 2015). Similarly, the quality of the social networks change over time with research highlighting that negative relationships with social supporters dominate the abusive and break‐up stages but shift towards more positive relationships in the longer term (Nolet et al., 2020). This means that interventions can employ a number of practices/activities to maximise opportunities to develop positive relationships and sources of social support over time (Goodman et al., 2016; Nolet et al., 2020). Interventions that enhance social support can serve to bolster victim‐survivors' well‐being and mediate the impact of abuse on a number of outcomes (Beeble et al., 2009; Goodman et al., 2005). Interventions that interrupt or target social isolation are also expected to support help‐seeking, serve as a protective factor against revictimisation and contribute to the process of change and long‐term recovery (Beeble et al., 2009; Goodman et al., 2005; Melgar et al., 2020; Zaphor et al., 2018).
(d) ISS interventions that target the community within which the informal social support takes place.
  Research identifies the growing importance of interventions that focus on community capacity building and collective efficacy in responding to DVA (Edwards et al., 2014; Sullivan, 2018). Such interventions recognise that there are multiple layers of social support and networks for victim‐survivors, with community organisations, neighbours and community members having an important role to play in identifying and responding to violence and abuse (Arón & Lorion, 2003).
  Akin to a ‘framework synthesis’ approach to systematic reviewing (Thomas et al., 2017), this framework will guide our approach to the EGM. This means that key steps in the process such as defining the inclusion criteria and the coding of studies will be aligned with this conceptual framework and how we expect ISS interventions to lead to positive outcomes for victim‐survivors of DVA.

### 3.5 Dimensions

#### 3.5.1 Types of study design

All study designs will be included in the EGM to understand the nature and extent of the primary empirical evidence on informal social support interventions. This ensures maximum identification and coverage of intervention research on informal social support interventions. To date, many domestic violence interventions that are well established as ways of responding to victim‐survivors (such as shelters and hotlines) do not have an underpinning evidence base. Some routine interventions have not been rigorously tested or formally evaluated (Bender, 2017) and so intervention studies in the field of DVA are relatively sparse. Experimental studies are particularly lacking (Feder et al., 2011). The focus on informal social support interventions which, by definition, sit outside formal agencies or services, means that rigorous evaluation is less likely to be mandated, required and/or sufficently funded. Systematic reviews of interventions delivered by the voluntary sector report a low number of studies, potentially explained by the lack of capacity and/or resources of this sector to carry out research (Konya et al., 2020). Therefore, this EGM seeks to be inclusive in terms of study design and eligible forms of research evidence.

For the methods of this EGM, this means:

1) Diverse study designs, reporting qualitative or quantitative data, will be included. As outlined above, sources of evidence that evaluate informal social support interventions are likely to be diverse in study design, method and type of data. Moreover, there is an established precedent for using qualitative and quantitative reports together in understanding the impacts of informal social support interventions (Konya et al., 2020; Ogbe et al., 2020). Strict limits in terms of methodological approach will not be applied because we know that intervention studies in the field of domestic violence face a number of challenges including small sample sizes and reliance on pilot interventions (Trabold et al., 2020). Eligible studies will therefore aim to assesses the impact/perception of impact of the intervention against the outcomes stipulated below. Reports will be excluded if they describe a programme/intervention but do not provide any data (qualitative or quantitative) that serves to evaluate the intervention (as outcomes or perceptions of outcomes).
  The study design of included studies will classified according to the categories outlined in Hong et al. (2018): Qualitative, Quantitative randomised controlled trials, Quantitative non‐randomised, Quantitative descriptive, Mixed Methods. Study design will be captured by the Data Extraction tool (see Supporting Information: Appendix 9).
  Eligible systematic reviews will not be directly included in the EGM but used as a search source to identify primary empirical studies.
2) Grey literature will be included.
  Informal social support interventions are likely to be delivered by and/or involve voluntary sector organisations, workplaces, and/or wider community organisations. Research associated with such interventions will therefore be reported in ‘grey’ literature sources alongside or instead of traditional academic channels. Grey literature includes ‘that which is produced on all levels of government, academics, business and industry in print and electronic formats, but which is not controlled by commercial publishers’. (cited in Tyndall, 2010). This will include dissertations, conference papers and research reports published by organisations in the field.

Evidence will only be included if the study is published in English to meet the rapid imperative of this project. Funded as part of the UK Research and Innovation (UKRI) response to the Covid‐19 pandemic, this project will use streamlined methods to complete the EGM in a relatively short period of time to satisfy extant need. Limiting the selection by language is one such common method (Tricco et al., 2015).

Ongoing studies will be excluded if there is no clear completion data/uncertainty about completion.

#### 3.5.2 Types of intervention/problem

The study must include an ISS intervention targeting DVA. Such interventions are defined as ‘Systematic activities designed to change the existing quality, level, or function of an individual's personal social network or to create new networks and relationships' (Budde & Schene, 2004; 342). To be eligible, the intervention needs to fit within the parameters of the following criteria.

*Type of ISS intervention*: The EGM recognises that ISS interventions are diverse, varying in their aims and nature. Interventions aim to create or enhance informal social support by targeting the providers of the informal social support, the victim‐survivor, or the relationship between them. Interventions can also aim to change the wider community within which informal social support is provided (as outlined in Conceptual Framework above).

Whilst a wide range of interventions may have an indirect effect on informal social support, this EGM is only interested in those interventions that specifically target informal social support. Therefore, to be included, interventions (or one component of them) need to have an explicit aim to enhance/promote informal social support. For example:

- Support groups for DVA victim‐survivors are only included if/when explicitly aiming to enhance informal social support via the intervention.
- Advocacy interventions are only included if they include an explicit aim to support the victim‐survivor to build or enhance their informal social networks.
- Community focused interventions are only included when they are tailored towards the informal social support relationship (e.g. training community members as advocates) rather than general community focused DVA interventions (e.g. general awareness raising campaigns).

*Provider of informal social support*: Informal social support interventions enable the provision of support to victim‐survivors from their friends, colleagues, neighbours or community members, current non‐abusive partners, or any family member. To do so, an intervention may serve to bolster the provision of *direct* support from victim‐survivors' existing networks and/or involve external actors in the facilitation of informal social support seeking. The EGM seeks to recognise the growing role for professional involvement in facilitating informal social support (e.g. Goodman et al., 2016) but this does not include the direct delivery of informal social support to the victim‐survivor of DVA. Interventions may include a role for professionals/practitioners in *facilitating* the victim‐survivor's ability to engage with their informal social supporters, for example, supporting the victim‐survivor to reach out and/or broaden their social networks. Interventions will be excluded if social support is directly provided by formal sources/professionals: services provided by the state, nongovernmental organisations/third sector and the legal system ‐ police, domestic abuse professionals, shelters, support workers, and counselling (Kelly, 1996). Similarly, this EGM recognises that eligible interventions may include payment for volunteers or professionals in their role as facilitating/promoting informal social support.

#### 3.5.3 Types of population

The study population must include (1) *Victim‐Survivors of DVA* AND/OR their (2) *Informal social supporters*

1) *Victim‐Survivors of DVA* (Any adults or young people who are/have been experiencing violence and abuse in a current or former intimate relationship). This EGM will focus on intimate partner violence, which is common in systematic reviews of interventions to address DVA (e.g., Anderson et al. (2021); Anderson et al., 2019; Rivas et al., 2016) and specifically social support (e.g. Gregory et al., 2017; Ogbe et al., 2020). This definition makes a conceptual distinction between family violence and abuse (e.g., sibling abuse or child‐to‐parent abuse) and intimate partner violence and abuse (abuse ‘by a current or former intimate partner', Ogbe et al., 2020). The definition of DVA is based on the British Home Office definition (2013): ‘Any incident or pattern of incidents of controlling, coercive, threatening behaviour, violence or abuse … The abuse can encompass, but is not limited to: psychological, physical, sexual, financial & emotional’. There are no restrictions on age, nationality or gender of the population. An exception to this, however, is that children who witness DVA (recognised as victims by the Domestic Abuse Act, 2021) will not be an eligible population group in this review. This is because the abusive relationship is not between intimate partners. This criterion means that there is a natural age restriction to the study.
2) *Informal social supporters* refers to friends, colleagues, neighbours or community members, current non‐abusive partners, any family member (including step‐family, non‐blood relatives, family‐in‐law) (developed from the Gregory et al., 2017 definition).

In a mixed sample (e.g., practitioners and victim‐survivors), the outcomes need to be reported separately for each population group (so findings for victim/survivors and/or informal supporters can be isolated).

#### 3.5.4 Types of outcome measures

This EGM will not use outcomes as eligibility criteria. In line with the Conceptual framework, the EGM will map the evidence against a broad range of outcome categories. These are defined in Table 1. This illustrates how different forms of data (qualitative and quantitative) can be coded against each outcome category. Table 1 includes examples of quantitative data instruments and forms of qualitative data coded against the outcomes. Qualitative outcomes will be derived from different levels of data, including quotes from participants (first order data), themes determined by authors of the primary study (second order data), or interpretation by review authors/coders (third order data). In addition to these outcome categories, we will be alert to other potentially relevant themes in the data. While the different order data have different levels of reliability, this is a recognised approach for meta‐ethnographic reviews of qualitative data (e.g., Sandelowski & Barroso, 2006).

**Table 1:** Outcome Categories

Category	Definition	Example quantitative instruments	Example qualitative data (1st, 2nd or 3rd order)
Cognitive	Knowledge or attitudes about DVA, including awareness of support and resources.	Domestic Violence Myths Acceptance Scale (DVMAS)	‘Program participants declared that the sessions they found most helpful involved content on: (a) safety planning, (b) community resources to address and obtain help with IPV, (c) information about the cycle of violence, and (d) information about the effects of IPV on women and children’. (2nd order data in Macy et al., 2012, p. 459)
Behavioural	Any behaviour or action including formal help‐seeking by victim‐survivor or provision of support by informal supporter	Decisional Conflict Scale	‘I'm still taking the help she gave me and using it on my own’ (1st order data in Allen et al., 2012, p. 12) Women who formerly faced gender‐based violence in silence began to call for help (3rd order data, Schuler et al., 2011)
Social network	The structure (size, density, composition), function (provision of support/response) or dynamics (relationship) of the victim‐survivor's social support network	The Interpersonal Support Self Evaluation List (ISEL)	‘Rejoining the community’ theme (2nd order data in Allen and Wozniak, 2010, p. 50)
Violence and Abuse	Experiences of violence and abuse	Severity of Violence Against Women Scale (SVWAS)	‘It's got a lot less toxic, basically’ (1st order data in Zakheim, 2009, p. 129)
Characteristics of relationship between victim‐survivor and perpetrator	Characteristics of the relationship, e.g. emotional involvement, intimacy, love, communication, decision‐making	The Attitudes Towards Marriage and the Family Scale	‘A woman during her interview said that she used to obey her husband and his family member's orders even for small issues like when to eat’ (2nd order data in Mahapatro & Singh, 2020, p. 287)
Mental health	Clinical/diagnosis of mental health such as depression, PTSD, anxiety.	PTSD Checklist	Women used coping strategies that served to protect their mental health (3rd order data in Mahapatro & Singh, 2020)
Psycho‐Social	Quality of life, well‐being and self‐efficacy	Domestic Violence Self‐Efficacy (DVSE) instrument	‘…it just makes me feel better and just gives me strength each time’ (1st order data in Taylor, 2000, p. 521)
Physical Health	Physical health	The Health Screening Questionnaire (HSQ)	
Employment or Education			
Economic			
Housing			
Parenting			

### 3.6 Search methods and sources

To ensure comprehensive coverage of academic and grey literature sources, a multi‐stranded search approach will be taken. This will include: Bibliographic databases, specialist DVA databases, policy‐orientated databases, systematic review databases and websites of DVA organisations. Citation checking of relevant systematic reviews and key primary studies will be undertaken. Key authors will also be contacted. Further details for each source are outlined below.

Bibliographic databases: Five sources will be used to search across disciplines relevant to DVA. A search string wa developed on the basis of terms used in similar reviews (Gregory et al., 2017; Ogbe et al., 2020). Databases will include APA PsycINFO via Ovid (search terms specified in Supporting Information: Appendix 2), Social Policy and Practice via Ovid (search terms in Supporting Information: Appendix 3), ASSIA via ProQuest (search terms specified in Supporting Information: Appendix 4), PubMed (search terms specified in Supporting Information: Appendix 5) and Social Science Citation Index via Web of Science (search terms specified in Supporting Information: Appendix 6).

Policy orientated databases: European Commission project databases, European Commission CORDIS database, World Health Organisation IRIS database (search terms specified in Supporting Information: Appendix 7).

Systematic review databases: Social Systems Evidence, Campbell Collaboration (search terms specified in Supporting Information: Appendix 7).

Specialist DVA databases: National Resource Centre on Domestic Violence, Anrows library, World Health Organisation violence against women database (search terms specified in Supporting Information: Appendix 8).

Websites of DVA organisations (search terms, where applicable, specified in Supporting Information: Appendix 8): SafeLives, Women's Aid, Imkaan, AVA, Respect, Standing Together, Galop, Surviving Economic Abuse, Refuge, Solace, Mankind, Sagesse, Domestic Violence Resource Centre Victoria, Loved Ones of Domestic Abuse Survivors, The Empowerment Network, Bambuuu, Bromley Well.

Snowballing techniques will also be employed to identify potentially eligible studies: identifying studies/intervention reports referenced by relevant systematic reviews, key excluded studies (e.g., studies that are highly relevant but do not report empirical data) or included studies.

Contacting key authors and stakeholders. Members of the advisory group will be asked for recommendations for studies, reports and/or evaluations. Key authors in the field of informal social support interventions will also be contacted to seek further potentially eligible studies.

### 3.7 Analysis and presentation

#### 3.7.1 Report structure

The results of the EGM will provide a descriptive overview of the nature and extent of the primary empirical research on informal social support interventions. This will be structured according to the main types of intervention (type, setting, provider of social support) study population (victim‐survivors, informal supporters, practitioners and/or communities, geography), study design, and outcomes (specified in Table 1).

The report will describe and analyse the spread and concentration of the evidence across the four types of intervention types. It will explore the type of outcomes that have been reported, including a consideration of the outcomes reported for victim‐survivors by their gender, ethnicity, age, exposure to abuse and migration status. The report will highlight evidence trends and the key gaps/limitations of the current evidence base. Policy, practice and research implications will be considered.

Figures will include the conceptual framework and flow of studies through the EGM. Tables will summarise included studies, by intervention type.

#### 3.7.2 Filters for presentation

The findings of the EGM will be presented as a matrix of intervention type and outcomes. Additional filters will include geographical region, study population (victim‐survivors or informal social supporters), and characteristics of informal support (setting, provider). The size of the bubble will reflect the number of studies and the colour of the bubbles will represent study design.

#### 3.7.3 Dependency

Each item in the map represents a single study. Where multiple reports exist for the same study, a main report will be identified and relevant reports will be linked (and used for descriptive purposes).

### 3.8 Data collection and analysis

#### 3.8.1 Screening and study selection

To develop shared understanding of the screening process, a team of four reviewers will initially screen a sample of titles and abstracts in pairs. Decisions will be discussed and discrepancies between pairs will be resolved by a third reviewer until a high level of consistency is reached (and inclusion/exclusion criteria are sufficiently clear). Individual reviewers will then work independently, with a process built in for identifying studies that require the judgement of a second reviewer. A sample of studies included on title and abstract will then be screened on full text by the four reviewers. Individual reviewers will then work independently, with a process built in for identifying studies that require the judgement of a second reviewer. Decisions on complex studies will be discussed by all four reviewers.

All references identifed in the search will be imported and screened using EPPI Reviewer software.

#### 3.8.2 Data extraction and management

All studies included on full text and subsequently in the EGM will be coded by two independent reviewers. Coding decisions will be discussed and agreed with a third reviewer serving as moderator, where necessary.

The EGM will include data extraction for: Study Population, Study, Intervention, Outcomes. For the complete tool see Supporting Information: Appendix 9. This tool will code the study design of included studies. Primary studies will not be quality appraised.

EPPI Reviewer software will be used for the process of data extraction, information management and production of the online evidence map.

#### 3.8.3 Tools for assessing risk of bias/study quality of included reviews

Systematic reviews will not be included in the EGM but are used as a source of primary studies.

#### 3.8.4 Methods for mapping

EPPI Reviewer Software will be used for the entire EGM process.

## CONTRIBUTIONS OF AUTHORS

- Content: Martha Tomlinson, Nicola Stokes, Carol Rivas, Karen Schucan Bird
- EGM methods: Karen Schucan Bird, Carol Rivas
- Statistical analysis: Karen Schucan Bird, Carol Rivas
- Information retrieval: Karen Schucan Bird, Nicola Stokes, Carol Rivas. Nicola Stokes

## DECLARATIONS OF INTEREST

Nicola Stokes and Martha Tomlinson are employed at SafeLives, a UK‐wide charity dedicated to ending domestic abuse.

Karen Schucan Bird and Carol Rivas have no conflicts of interest with respect to the content of the EGM.

## SOURCES OF SUPPORT

**Internal sources**

- No internal sources of support.

**External sources**

Economic and Social Research Council, UK

This study is funded by the Economic & Social Research Council (ESRC), as part of UK Research & Innovation's rapid response to Covid‐19.

## Supporting information

Supporting information.
