# Supplementary material for: PROTOCOL: Informal social support interventions for improving outcomes for victim‐survivors of domestic violence and abuse: An evidence and gap map
Source: Campbell Syst Rev. 2022 Jun 30;18(3):e1263. doi: 10.1002/cl2.1263 (PMC9246292; doi:10.1002/cl2.1263)
Supplement: Supplementary file 1 — Supporting information. [file CL2-18-e1263-s001.docx]

# Appendices

## 1 Link to online interactive EGM

Add link at the full report stage

## 2 Search terms APA PsychInfo via Ovid

| ((support system or social support or community support or social network or community network or family support or family involvement or parental support or community involvement or friend support or workplace support or informal social support or peer support) and (domestic violence or partner violence or battered woman or spouse abuse or spousal abuse or ipv or battered or abuses or abusive or violent or pregnant women or domestic abuse or intimate partner violence or partner abuse)).mp. |
| --- |

## 3 Search terms for Social Policy and Practice via Ovid

| ((support system or social support or community support or social network or community network or family support or family involvement or parental support or community involvement or friend support or workplace support or informal social support or peer support) and (domestic violence or partner violence or battered woman or spouse abuse or spousal abuse or ipv or battered or abuses or abusive or violent or pregnant women or domestic abuse or intimate partner violence or partner abuse)).ab. |
| --- |

## 4 Search terms for ASSIA via Proquest

ADD

## 5 Search terms for Pub Med

"domestic violence"[MeSH Terms] OR ("domestic"[All Fields] AND "violence"[All Fields]) OR "domestic violence"[All Fields] OR (("partner"[All Fields] OR "partner s"[All Fields] OR "partnered"[All Fields] OR "partnering"[All Fields] OR "partners"[All Fields]) AND ("violence"[MeSH Terms] OR "violence"[All Fields] OR "violence s"[All Fields] OR "violences"[All Fields])) OR ("battered women"[MeSH Terms] OR ("battered"[All Fields] AND "women"[All Fields]) OR "battered women"[All Fields] OR ("battered"[All Fields] AND "woman"[All Fields]) OR "battered woman"[All Fields]) OR ("spouse abuse"[MeSH Terms] OR ("spouse"[All Fields] AND "abuse"[All Fields]) OR "spouse abuse"[All Fields] OR ("spousal"[All Fields] AND "abuse"[All Fields]) OR "spousal abuse"[All Fields]) OR (("domestic"[All Fields] OR "domestically"[All Fields] OR "domesticate"[All Fields] OR "domesticated"[All Fields] OR "domesticates"[All Fields] OR "domesticating"[All Fields] OR "domestication"[MeSH Terms] OR "domestication"[All Fields] OR "domestications"[All Fields] OR "domestics"[All Fields]) AND ("abusable"[All Fields] OR "abuse s"[All Fields] OR "abused"[All Fields] OR "abuser"[All Fields] OR "abuser s"[All Fields] OR "abusers"[All Fields] OR "abuses"[All Fields] OR "abusing"[All Fields] OR "abusive"[All Fields] OR "abusively"[All Fields] OR "abusiveness"[All Fields] OR "abuse"[All Fields])) OR "ipv"[All Fields] OR ("intimate partner violence"[MeSH Terms] OR ("intimate"[All Fields] AND "partner"[All Fields] AND "violence"[All Fields]) OR "intimate partner violence"[All Fields]) OR ("intimate partner violence"[MeSH Terms] OR ("intimate"[All Fields] AND "partner"[All Fields] AND "violence"[All Fields]) OR "intimate partner violence"[All Fields] OR ("intimate"[All Fields] AND "partner"[All Fields] AND "abuse"[All Fields]) OR "intimate partner abuse"[All Fields]) OR ("spouse abuse"[MeSH Terms] OR ("spouse"[All Fields] AND "abuse"[All Fields]) OR "spouse abuse"[All Fields] OR ("partner"[All Fields] AND "abuse"[All Fields]) OR "partner abuse"[All Fields])

AND

"community networks"[MeSH Terms] OR "psychosocial support systems"[MeSH Terms] OR "social networking"[MeSH Terms] OR "social networking"[MeSH Terms] OR "social support"[Text Word] OR "social network"[Text Word] OR "social system"[Text Word] OR "informal social support"[Text Word]

## 6 Search terms for SSCI

TS=(domestic violence or partner violence or spouse abuse or domestic abuse or intimate partner violence or intimate partner abuse or partner abuse)

AND

TS=(support system or social support or community support or social network or community network or family support or family involvement or parental support or community involvement or friend support or workplace support or informal social support or peer support)

## 7 Search terms for systematic review and policy-orientated databases

"domestic abuse" OR "domestic violence" OR "partner violence" OR "partner abuse" OR "spousal abuse" OR "spouse abuse" AND “informal social support” OR “informal support” OR “community support” OR “family support” OR “friends support” OR “workplace support” OR “community network” OR “support system” OR “friends and family” OR “peer support” OR “social network” OR “social networks” OR “social support”

## 8 Search terms for DVA specialist databases and websites

 “community network” OR “community support” OR “family support” OR “friends support” OR “friends and family” OR “informal support” OR “peer support” OR “social network” OR “social networks” OR “social support” OR “support system” OR “workplace support”

## 9 Data Extraction tool

**Mapping**

- 1. Study Population
- *Select all that apply*
  - Victim-Survivors *Victim-Survivors of Domestic Violence and Abuse (Any adult or young people who are/ have been experiencing violence and abuse in a current or former intimate relationship).*
  - Exposure to DVA? (at time of study)
    - In abusive relationship
    - No longer in abusive relationship (short term) *Up to 1 year*
    - No longer in abusive relationship (Long term) *Over 1 year*
    - Not reported
- What gender?
  - Female
  - Male
  - Non-binary
  - Not reported
- What ethnicity/ race?
  - Ethnic/ racial minority
  - Ethnic/ racial majority
  - Not reported
- What age?
  - Adults
  - *18 years old and upwards*
  - Young people and Adolescents *Under 18 years*
  - Not reported
  - Migrated population *Select if the population includes individuals/ groups who are identified/ recognised as immigrant populations*
- Informal Supporters
- *Informal social supporters refers to friends, colleagues, neighbours or community members, current non-abusive partners, any family member (including step-family, non-blood relatives, family-in-law) (developed from Gregory et al., 2017 definition).*
  - What type of informal supporter?
- *Sample designed to capture data from these informal supporters*
  - Peer *Peer: shared personal experience (Nesta, 2015).*
  - Friend
  - Family member
  - Colleague
  - Faith leaders *Clergy, religious leaders*
  - Community member
  - neighbour
  - Other (please specify)
  - Not reported
  - Practitioners *Practitioners from formal services in state, non-governmental organisations/ third sector and the legal system -police, professional workers, shelters, support workers, and counsellors (Kelly et al, 1996).*
  - Communities *Community wide populations (general level population groups that are likely to include range of informal supporters and victim/survivors)*
- 2. Study
  - What was the aim of the study?
    - Explicitly reported (specify)
    - Implicit (specify)
  - What country was study/ intervention set in?
    - UK
    - USA
    - Uganda
    - China
    - Hong Kong
    - Unspecified (online)
    - Canada
    - Portugal
    - Denmark
    - South Africa
    - Australia
    - Sweden
    - Vietnam
    - Mexico
    - Not reported
    - ADD (please specify)
    - Rwanda
    - India
    - Netherlands
    - global
  - What type of data/ study?
    - Qualitative
    - Quantitative
  - What was the study design?
    - Quantitative Randomised Control Trial *A study in which ‘individuals are allocated to intervention or control groups by randomization’*
    - Quantitative non-randomised studies *Any quantitative studies estimating the effectiveness of an intervention, with single or multiple groups, that do not use randomization to allocate individuals to groups. Such as quasi-experimental studies, Before and After study,*
    - Quantitative descriptive studies *‘describe the existing distribution of variables’, non-comparative study such as survey, descriptive cross-sectional study*
    - Qualitative studies *Qualitative data collection and analysis. Such as interview, focus group, case study.*
    - Mixed Method studies *A study that combines qualitative and quantitative methods (as separate components that are integrated in the analysis).*
- 3. Informal Social Support Intervention
  - What is the informal social support intervention?
    - Education/ training
    - Community activities
    - Support group
    - Online forum
    - Advocacy (with explicit ISS element)
    - Provision of information (e.g. posters, leaflets)
    - Policy development
    - Other (specify)
    - Community based groups *This refers to groups of community members/ individuals who come together to discuss and/ or action issues related to DVA*
    - Pyscho-therapy (with explicit ISS element)
    - Informal adjudication methods T*his includes restorative justice groups or other methods to adjudicate between victim-survivor and perpetrator.*
- What type of intervention? Select one
  - a) Targetting the provider of informal social support *ISS interventions that focus on the informal supporters and seek to mobilize existing social support and/ or develop new relationships and mechanisms for support.*
  - b) Targetting how the support is provided *ISS interventions shape the quality of support and/ or relationship between informal support and DVA victim-survivor.*
  - c) Targetting victim-survivors ability to engage with ISS *ISS interventions that focus on victim-survivors’ ability to engage with, and utilise, informal social support*
  - d) Targetting the community in which ISS takes palce *Communities are both physical places/ spaces and forms of social organization within which informal support operates (Mancini et al., 2006)*
  - Other
- Where the informal social support will be/was delivered?
  - Home
  - Online
  - Shelter
  - Workplace
  - Education
  - Place of Worship
  - Wider community
  - Other (please specify)
  - Not reported
  - Nonprofit organization
- Who will provide/provided the informal social support?
  - What type of informal supporter? *Sample designed to capture data from these informal supporters*
  - Peer
  - Friend
  - Family member
  - Colleague
  - Faith leaders, *Clergy, religious leaders*
  - Other (please specify)
  - Not reported
  - Community member
- Do they share common language/ culture with victim-survivors?
  - Yes
  - No
  - Not reported
- Prior experience of DVA?
  - Yes, as victim-survivor
  - Yes, as practitioner/ professional
  - No reported experience
  - Not reported
- Have they recieved training to provide informal support?
  - Yes
  - No
  - Not reported
- Multi-component intervention*The enhancement or provision of informal Social Support is only one aspect of an intervention with multiple elements.*
- 4. Main outcomes/ study tells us about...
  - Victim-Survivors
    - Cognitive (knowledge or attitudes) *Knowledge or attitudes about DVA*
    - Awareness, knowledge and understanding of DVA
    - Knowledge of support resources
  - Behavioural *Outcomes relating to victim-survivor's actions to stay in relationship and/ or seek help, obtain support.*
    - Accessing/ obtaining informal social support
    - Informal help seeking *e.g. new or extended help seeking from informal sources*
    - Formal help seeking *e.g. calls to police*
    - Ongoing involvement with abusive partner
    - Disclosure of DVA *e.g. disclosure of DVA to informal or formal supporter (i.e. friend, family, neighbours, religious leader, or professional, practitioner in DVA or health sector)*
    - Readiness to change DVA
    - Social network *Any outcomes/ data reporting on the structure (size, density, composition), function (provision of support/ response) and dynamics (relationship) of the victim-survivor's social support network*
    - Size or strength of informal social network
    - Violence or abuse *Experiences or incidences of any form of DVA, reports of abuse or violence.*
    - Characteristics of relationship between victim-survivor and perpetrator
    - Psychosocial outcomes *e.g.life satisfaction, social connectedness, self efficacy*
    - Domestic violence self efficacy *i.e. self confidence in managing abuse-related difficulties, solving problems, and helping oneself (measure used in Ross, 2013, see p. 38)*
    - Assertiveness
    - Satisfaction with informal social support
    - Mental health outcomes *e.g.depression, anxiety, self-esteem*
    - Physical health outcomes
    - Employment or education outcomes
    - Economic outcomes
    - Parenting outcomes
    - Housing outcomes
- Informal Social Supporters
  - Cognitive
    - Awareness and Understanding of DVA
    - Knowledge of support resources
    - Confidence in providing ISS
  - Behavioural
    - Provision of informal support
  - Psychosocial outcomes *e.g. perception of social support, Quality of Life, self-esteem*
  - Other (please specify)
- Community level
  - Awareness and understanding of DVA *Recognition of intimate partner violence and abuse; different types*
  - Social acceptance of DVA *Acceptability of DVA in a community or society.*
  - Provision of informal social (community) support
  - Confidence in providing ISS
